# Correction: Jiang et al. Characterization of Functional Microorganisms in Representative Traditional Fermented Dongcai from Different Regions of China. *Foods* 2023, *12*, 1753

**DOI:** 10.3390/foods13193190

**Published:** 2024-10-08

**Authors:** Yanbing Jiang, Hao Fu, Meng Li, Changtao Wang

**Affiliations:** 1 Beijing Key Laboratory of Plant Resource Research and Development, College of Chemistry and Materials Engineering, Beijing Technology and Business University, Beijing 100040, China; 2 Institute of Cosmetic Regulatory Science, Beijing Technology and Business University, Beijing 100040, China

There was an error in the original publication [1]. A correction has been made to reference [32]. The updated reference is as follows:

32. Lee, M.; Song, J.H.; Jung, M.Y.; Lee, S.H.; Chang, J.Y. Large-scale targeted metagenomics analysis of bacterial ecological changes in 88 kimchi samples during fermentation. *Food Microbiol*. **2017**, *66*, 173–183.

The authors state that the scientific conclusions are unaffected. This correction was approved by the Academic Editor. The original publication has also been updated.
